# Multifaceted Biological Properties of Verbascoside/Acteoside: Antimicrobial, Cytotoxic, Anti-Inflammatory, and Immunomodulatory Effects

**DOI:** 10.3390/antibiotics14070697

**Published:** 2025-07-11

**Authors:** Mirjana Marčetić, Biljana Bufan, Milica Drobac, Jelena Antić Stanković, Nevena Arsenović Ranin, Marina T. Milenković, Dragana D. Božić

**Affiliations:** 1 Department of Pharmacognosy, Faculty of Pharmacy, University of Belgrade, Vojvode Stepe 450, 11221 Belgrade, Serbia; milica.drobac@pharmacy.bg.ac.rs; 2 Department of Microbiology and Immunology, Faculty of Pharmacy, University of Belgrade, Vojvode Stepe 450, 11221 Belgrade, Serbia; biljana.bufan@pharmacy.bg.ac.rs (B.B.); jelena.stankovic@pharmacy.bg.ac.rs (J.A.S.); nevena.arsenovic-ranin@pharmacy.bg.ac.rs (N.A.R.); marina.milenkovic@pharmacy.bg.ac.rs (M.T.M.); dragana.bozic@pharmacy.bg.ac.rs (D.D.B.)

**Keywords:** verbascoside, acteoside, cytotoxicity, antimicrobial effect, anti-inflammatory effect, immunomodulatory effect

## Abstract

Verbascoside is a polyphenolic compound that belongs to the phenylethanoid glucosides. It occurs in more than 220 plant species. The species with high content of this compound are used in folk medicine, and in modern phytotherapy, mostly based on its recognized anti-inflammatory and antimicrobial effects. Studies conducted so far confirmed these effects, and also pointed to others (i.e., anti-cancer, neuro-, cardio-, hepato-, and nephro-protective). This review presents data on the chemistry, occurrence, and biosynthesis of verbascoside. Additionally, it focuses on the cytotoxic, antimicrobial, anti-inflammatory, and immunomodulatory effects, as well as the main cellular and molecular mechanisms of its action.

## 1. Introduction

Verbascoside, also known as acteoside, kusaginin, or orobanchin, is a plant secondary metabolite that is widely distributed in various plant species. It exhibits a wide range of biological activities, such as antioxidant, anti-inflammatory, anti-diabetic, cardiovascular, and hepatoprotective properties. In addition, verbascoside has shown neuroprotective effects that could be helpful in the treatment of neurological diseases such as Alzheimer’s and Parkinson’s [1,2,3].

Verbascoside can be isolated from medicinal plants commonly used in folk medicine, such as *Verbascum thapsus* (common mullein), *Verbena officinalis* (common vervain), *Plantago lanceolata*, and *Olea europaea* (olive). Due to its antimicrobial and anti-inflammatory properties, it has been traditionally used for the treatment of infections, wounds, and inflammatory conditions [4]. Its antimicrobial properties are particularly noteworthy, as it is effective against a range of bacterial pathogens, including multidrug-resistant strains, as well as viruses, protozoa, and fungi [2,5,6]. This makes it a promising candidate for the development of new antimicrobial agents in the face of increasing antimicrobial resistance. Verbascoside’s anti-inflammatory properties are also important, as inflammation is a common factor in many chronic diseases. Verbascoside inhibits the production of pro-inflammatory cytokines and mediators, offering potential therapeutic benefits in conditions such as arthritis and inflammatory bowel disease [1,2]. Verbascoside also has immunomodulatory effects that may alter the body’s immune response. This property is particularly important in the context of infectious diseases and autoimmune disorders, where modulation of the immune system can lead to better treatment of the disease [1,2,3]. In addition to its antimicrobial activity, verbascoside shows cytotoxic effects against various cancer cell lines, suggesting its potential use in cancer therapy. Studies have shown that it is able to induce apoptosis and inhibit cell proliferation, making it a valuable compound for further research in oncology [7].

This comprehensive review article aims to evaluate and summarize the current scientific knowledge on the antimicrobial, cytotoxic, anti-inflammatory, and immunomodulatory activities of verbascoside. By examining in vitro and in vivo studies, the mechanisms underlying these biological activities will be elucidated and the importance of verbascoside’s multifaceted properties in combating microbial resistance, inflammation, and immune-related diseases will be emphasized, thereby contributing to the development of novel treatment strategies. A comprehensive search of the PubMed database was performed using the following keywords: “verbascoside”, “acteoside”, “kusaginin”, “orobanchin”, “biosynthesis”, “cytotoxicity”, “antimicrobial”, “antibacterial”, “antifungal”, “antiprotozoal”, “antiviral”, “immunomodulatory”, “anti-inflammatory”. The identified literature served as the basis for this review.

## 2. Chemistry and Occurrence of Verbascoside

**Figure 1:**
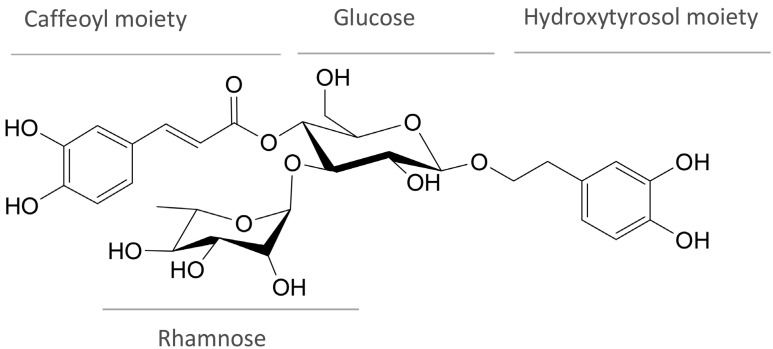
Structure of verbascoside.

Verbascoside [CAS number 61276-17-3, β-(3,4-dihydroxyphenylethyl)-O-α-L-rhamnopyranosyl-(1→3)-β-D-(4-O-caffeoyl)-glucopyranoside] is a polyphenolic compound that belongs to the class of phenylethanoid glucosides. Structurally, it consists of caffeic acid (3,4-dihydroxycinnamic acid), bound to glucose in the C4 position via an ester bond, the glucose being a part of disaccharide that also contains rhamnose, and hydroxytyrosol (4,5-hydroxyphenylethanol) bound to glucose in the position C1 via an ether bond (Figure 1) [8].

Verbascoside was first isolated from *Verbascum sinuatum* L. (Scrophulariaceae) in 1963 [9]. On the other hand, Birkofer et al. (1968) isolated acteoside from *Syringa vulgaris* L. (Oleaceae) [10], which was later found to be structurally identical to the previously isolated verbascoside. In addition, Sakurai and Kato (1983) reported the isolation of a new glycoside called kusaginin from *Clerodendron trichotomum* Thunb. (Lamiaceae) [11] whose structure was found to be identical to that of verbascoside. Although it was proposed to accept only one name [8], both names, verbascoside and acteoside, are used in the literature today, while the names kusaginin and orobanchin are less common.

Verascoside is widely distributed in nature; it occurs in more than 220 species, particularly in the plant families of the order Lamiales, although it has also been found in plants of the orders Asterales (families Asteraceae and Campanulaceae), Cucurbitales (family Cucurbitaceae), and Magnoliales (family Magnoliaceae) [1]. In the order Lamiales, it has been isolated from many plant species of the families Verbenaceae, Lamiaceae, Scrophulariaceae, Acanthaceae, and others, given in Supplementary Table S1. Verbascoside is most commonly isolated from the aerial parts, but there are also data on its isolation from roots/rhizomes or barks (Supplementary Table S1 [12,13,14,15,16,17,18,19,20,21,22,23,24,25,26,27,28,29,30,31,32,33,34,35,36,37,38,39,40,41,42,43,44,45,46,47,48,49,50,51,52,53,54,55,56,57,58,59,60,61,62,63,64,65,66,67,68,69,70,71,72,73,74,75,76,77,78,79,80,81,82,83,84,85,86,87,88,89,90,91,92,93,94,95,96,97,98,99,100,101,102,103,104,105,106,107,108,109,110,111,112,113,114,115,116,117,118,119,120,121,122,123,124,125,126,127,128,129,130,131,132,133,134,135,136,137,138,139]). Verbascoside plays a role in the plant’s defence system, protecting it from herbivores and UV radiation [140,141].

**Figure 2:**
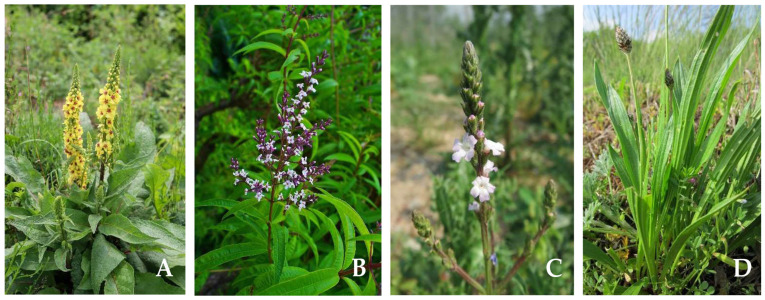
Plant species containing verbascoside. (**A**)—*Verbascum thapsus*, Pixabay (Pixabay License, https://pixabay.com/photos/mullein-nature-botany-6902726/ (accessed on 8 July 2025)); (**B**)—*Aloysia citriodora* (syn. *Lippia citriodora*) (Martín Vicente, CC BY 4.0, https://www.flickr.com/photos/martius/49694572733/ (accessed on 8 July 2025)); (**C**)—*Verbena officinalis* (Andreas Rockstein, CC0 1.0, https://commons.wikimedia.org/wiki/File:20150606Verbena_officinalis1.jpg (accessed on 8 July 2025)); (**D**)—*Plantago lanceolata* (CC0 1.0 Universal, https://commons.wikimedia.org/wiki/File:20130429Plantago_lanceolata.jpg (accessed on 8 July 2025)).

Verbascoside is a key constituent of many herbal drugs used in modern phytotherapy, such as mullein flower (*Verbasci flos*), lemon verbena leaf (*Verbenae citriodorae folium*), verbena herb (*Verbenae herba*), ribwort plantain (*Plantaginis lanceolatae folium*) (Figure 2), white horehound (*Marrubii herba*), devil’s claw root (*Harpagophyti radix*), ash leaf (*Fraxini folium*) [142], black horehound (*Ballotae nigrae herba*) [143]. In addition, verbascoside may also have a chemotaxonomic significance [144,145].

## 3. Biosynthesis of Verbacoside

One of the hot spots of the plant’s secondary metabolism is research on the molecular mechanism. Some biosynthetic pathways are completely defined, and some are still unclear. Verbascoside belongs to phenylethanoid glycosides that are made of monosaccharides, phenylethanol (C6–C2 skeleton with aldohexose/aldopentose attached by a glycosidic bond), and organic acids, such as caffeic acid, ferulic acid, and coumaric acid [146,147]. It is among the most widespread of the disaccharide caffeoyl esters [1] and is also one of the best-known phenylethanoids because of its health benefits [148].

Verbascoside (C_29_H_36_O_15_, MW 624.6 g/mol) is a water-soluble disaccharide derivative related to trans-caffeic acid and hydroxytyrosol [1]. It consists of four components: caffeic acid, glucose, rhamnose, and hydroxytyrosol (Figure 1) [146]. Investigation of biosynthetic pathways revealed that the hydroxytyrosol moiety is synthesized from tyrosine by the shikimate pathway, while the caffeoyl moiety is derived from phenylalanine by the cinnamate pathway [3].

**Figure 3:**
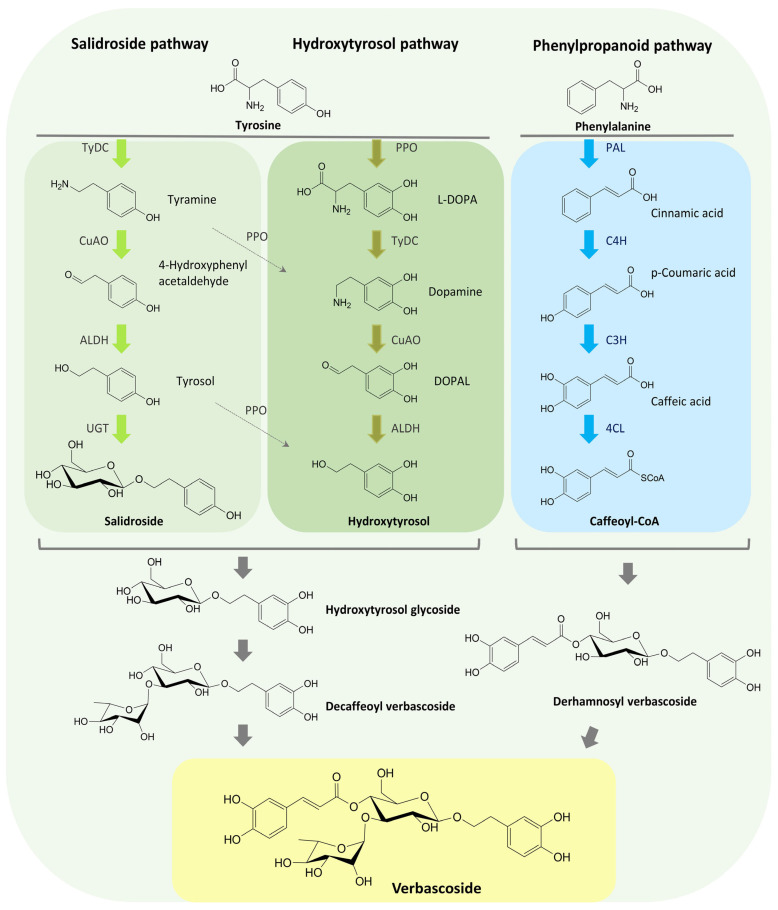
The different pathways of verbascoside biosynthesis. TyDC—tyrosine decarboxylase; CuAO—copper amine oxidase; ALDH—alcohol dehydrogenase; UGT—UDP-glucuronosyltransferase; PPO—polyphenol oxidase; PAL—phenyl ammonia lyase; C4H—cinnamate 4-hydroxylase; C3H—coumarate 3-hydroxylase; 4CL—4-coumarate CoA ligase [146,147].

The verbascoside biosynthesis can be divided into the upstream and downstream pathways (Figure 3). It begins with the phenylalanine and tyrosine generated by the shikimate pathway (upstream pathway) [146]. The shikimate pathway is the metabolic process that links the metabolism of carbohydrates to the biosynthesis of aromatic compounds. It is a seven-step process in which phosphoenolpyruvate and erythrose 4-phosphate are converted into shikimate and ultimately chorismate, the precursor of the aromatic amino acids like phenylalanine and tyrosine. The shikimate pathway is found only in microorganisms and plants, but not in animals or humans [149,150].

The upstream shikimate pathway is common to many natural products and is less complicated to elucidate than the specific downstream verbascoside pathway. This downstream pathway can be divided into four branches: (1) salidroside pathway from tyrosine to salidroside, (2) phenylpropanoid pathway from phenylalanine to caffeic acid, (3) hydroxytyrosol pathway from tyrosine to hydroxytyrosol, (4) some cross pathways among the first three ones [146].

The first branch, the salidroside pathway of verbascoside biosynthesis, is a putative pathway from tyrosine to tyramine, tyrosol (4-hydroxyphenethyl alcohol), and salidroside. Other identified intermediates are 4-hydroxyphenylacetaldehyde and p-hydroxyphenylacetone.

Enzymes and their corresponding genes have also been identified, namely tyrosine decarboxylase (TyDC), monoamine oxidase gene (MAO), 4-hydroxyphenyl acetaldehyde reductase gene (4HPAR), 4-hydroxyphenyl acetaldehyde synthetase gene (4HPAAS), tyrosine aminotransferase gene (TAT), UDP-glycosyltransferase (UGT) and tyrosinase (TYR) [146].

The second, phenylpropanoid pathway starts with phenylalanine and ends with caffeic acid or caffeoyl-CoA. This pathway is a part of the flavonoid and lignin biosynthetic pathway and has been extensively studied among plants [146,151]. Its intermediates, cinnamic acid, *p*-coumaric acid, *p*-coumaric acid-CoA, and caffeic acid, as well as enzymes phenyl ammonia lyase (PAL), cinnamate hydroxylase (C4H), 4-coumaric acid coenzyme A ligase (4CL), coumaric acid-3-hydroxylase (C3H), quinine hydroxycinnamyl transferase (HCT), tyrosine aminotransferase (TAT), and caffeoyl shikimate esterase (CSE), are currently accepted by researchers [146].

The third branch of verbascoside biosynthesis is the hydroxytyrosol pathway. Tyrosine can be converted to tyramine (salidroside pathway) as well as to L-DOPA (L-3,4-dihydroxyphenylalanine) by tyrosine hydroxylase or tyrosine 3-monooxygenase (TH). Further, L-DOPA gives dopamine, catalyzed by 3,4-dihydroxyphenylalanine decarboxylase (DDC). The biosynthesis of dopamine from tyrosine is also present in mammals. Hydroxytyrosol can be hydroxylated from tyrosol by tyrosine hydroxylase (TH) via the salidroside pathway. Another way of producing hydroxytyrosol is from dopamine, which can be oxidized to 3,4-dihydroxyphenyl-acetaldehyde (3,4-HPAA) by tyramine oxidase (TYO), and then reduced to hydroxytyrosol by alcohol dehydrogenase (ADH) [146,152].

Finally, the cross pathways are composed of several branch pathways from caffeic acid, hydroxytyrosol, and salidroside to verbascoside and enable the connection among the different pathways. That interrelation is based on the biosynthetic enzymes that can catalyse multiple products [146,152].

The verbascoside biosynthetic pathways, intermediates, and enzymes are mostly well defined and identified, but some of them are still putative. The phenylpropanoid pathway from phenylalanine to caffeoyl-CoA is common in flavonoid and lignin biosynthesis, and the enzyme genes involved in this pathway have been extensively studied. On the other hand, the genes encoding the enzymes responsible for the formation of hydroxytyrosol from tyrosine (copper-containing amine oxidase, CuAO, and alcohol dehydrogenase, ALDH) are putative and still not identified [151].

## 4. Cytotoxicity of Verbacoside

Testing the cytotoxicity of verbascoside both in vitro and in vivo is crucial for understanding its potential therapeutic applications and safety profile [3]. To evaluate its cytotoxicity towards normal cells and its anti-cancer properties, various mammalian cell lines are frequently used for in vitro studies, such as normal human embryonic diploid lung fibroblasts (IMR90 cells) and cancer cell lines such as the human breast cancer cell lines (MCF-7, MDA-MB-231), human osteosarcoma cell lines (U2 OS, Sa OS), or mouse cell lines [7,153]. These studies help to determine the ability of verbascoside to induce apoptosis and inhibit cell proliferation. In vivo studies usually involve animal models such as Wistar rats to evaluate the overall toxicity, bioavailability, and therapeutic efficacy of the drug [107]. These comprehensive evaluations are essential for the further development of verbascoside as a potential therapeutic agent.

In vitro studies on the cytotoxicity of verbascoside have shown that verbascoside has no cytotoxic effect on most of the cell lines tested. Low concentrations of verbascoside (0.78 μM) had no effect on the survival rates of the hepatoblastoma cell line HepG2, the human embryonic kidney cells HEK 293, and the adenocarcinomic human alveolar basal epithelial cells A549 (survival rates 90.24%, 82.06% and 84.38%). When higher concentrations (200 μM) were applied, the survival rates of HepG2 and HEK 293 were still high (87.19% and 78.72%), but this concentration showed low but acceptable cytotoxicity in A549 cells with a survival rate of 66.13% [154]. No cytotoxic effects were also observed in HepG2 and NIH cells at concentrations of up to 400 μM [155]. In primary lymphocyte cultures, verbascoside did not induce cytotoxicity or a significant increase in apoptosis when applied at concentrations ranging from 1.25 μM to 160 μM. In addition, treatment with 80 μM verbascoside induced the highest cell proliferation rate [156].

A study by Cheimonidi et al. reported the selective cytotoxicity of verbascoside isolated from the dried leaves of *Lippia citriodora* (Lamiaceae) [7]. The cytotoxicity was tested against several mammalian tumour cell lines (B16 melanoma mouse cells, YAC-1, WEHI-164 mouse cell lines, mouse skin cancer cell lines C5N and A5, and human osteosarcoma cell lines U2 OS and Sa OS), as well as against normal human embryonic diploid lung fibroblasts (IMR90 cells). Verbascoside had no cytotoxic effect on IMR90 cells after three weeks of exposure to 100 μΜ verbascoside, but instead showed a stimulatory effect on cell growth and delayed the progression of cellular senescence. Furthermore, verbascoside showed no toxicity when applied in vivo in Wistar rats or in *Drosophila melanogaster*. Conversely, the same concentration of verbascoside applied to human fibroblasts (100 μΜ) showed potent toxicity in mouse and human cancer cell lines after 24 h of exposure in vitro, and verbascoside suppressed tumour growth in vivo in the melanoma mouse model in C57BL/6 mice. The antitumour effect of verbascoside was achieved through changes in intracellular phosphorylation in cancer cells and immune response-related signalling pathways, leading to the activation of the anti-tumour immune response [7,157]. The cytotoxic effect of verbascoside was also reported for human breast cancer cell lines MCF-7 and MDA-MB-23, where the highest cytotoxic effect of 100 μM verbascoside was achieved after 24, 48, and 72 h of exposure [153]. On the other hand, verbascoside isolated from the aerial parts of *Leucophyllum frutescens* tested on the malignant cell lines HeLa (human cervical adenocarcinoma cells) and normal human cells MeT-5A (human mesothelial cells), showed no cytotoxic effect in the range of 10 to 500 μM. Accordingly, no acute cytotoxicity was observed in Wistar rats after oral administration of verbascoside and a 14-day follow-up, and the median lethal dose (LD_50_) was above 5000 mg/kg [107]. In vivo administration of verbascoside to BALB/c mice or Wistar rats up to 5000 mg/kg via the intraperitoneal or oral route also did not result in acute or subacute toxicity [155,158].

## 5. Antimicrobial Activity of Verbacoside

In recent years, numerous strategies have been researched to combat microbial resistance to conventional antimicrobial therapies. A particular focus has been on natural compounds, especially polyphenols, which are novel molecules with significant antimicrobial activities. In addition, natural compounds act synergistically on various antimicrobial intracellular targets, making the development of resistance more difficult. Verbascoside exhibits a broad spectrum of antimicrobial activities, including antibacterial, antifungal, antiprotozoal, and antiviral properties. In addition to direct cytocidal activities, it also acts on virulence factors of microorganisms and prevents the adhesion of microorganisms to the surface of host cells, their invasion, or the formation of biofilms [3]. Previous studies have shown that verbascoside is effective against microorganisms such as *Staphylococcus aureus*, *Escherichia coli*, *Klebsiella pneumoniae*, *Pseudomonas* spp., and various viruses, as well as fungi such as *Candida* spp., *Cryptococcus* spp., and *Aspergillus* spp. and the protozoa *Leishmania* spp. [2,5,6].

### 5.1. Antibacterial Activity

The resistance of bacteria to antimicrobial agents is a growing global health problem and poses significant challenges for the treatment of infectious diseases. Although antibacterial agents form the largest group of antibiotics with different classes and numerous members, most bacteria have already developed resistance through several important mechanisms, such as the production of enzymes that degrade or modify antibiotics, the modification of target molecules that reduce antibiotic binding, efflux pumps that expel antibiotics from the cell, and the reduction of membrane permeability that prevents antibiotic entry [159]. These mechanisms allow bacteria to survive and multiply despite antibiotic treatment, leading to prolonged infections, higher healthcare costs, and increased mortality rates. On the other hand, major components isolated from plants often act on multiple bacterial targets simultaneously, making the development of resistance less likely [154]. In addition, natural compounds have synergistic effects with conventional antimicrobials, reducing the need for high-dose and prolonged antibiotic therapy, which in turn reduces the likelihood of developing antimicrobial resistance [160].

The antimicrobial activity of verbascoside or extracts isolated from verbascoside-rich plants has already been investigated. Verbascoside isolated from *Stachytarpheta cayennensis* (Rich.) Vahl, Verbenaceae was tested against several bacterial strains using the agar diffusion method (results expressed as diameter of inhibition zone in mm) and the broth microdilution method to determine the minimum inhibitory concentration (MIC) of verbascoside that inhibits the growth of selected microorganisms (results expressed in μg/mL). Verbascoside exhibited a moderate antimicrobial activity against *Streptococcus pyogenes* (20 mm; 62 µg/mL), *Staphylococcus aureus* (13 mm; 63 µg/mL), and *S. epidermidis* (20 mm; 32 µg/mL) [161]. The extracts of plants from the Buddlejaceae family, which are endemic to Asia, Africa, and America, show strong to moderate antimicrobial activity against Gram-positive and Gram-negative bacteria with different MIC values depending on the *Buddleja* species and the extraction method. Verbascoside isolated from the leaves of *Buddleja salviifolia* (L.) Lam. showed good activity against *S. aureus* and *Klebsiella pneumoniae* with a MIC of 62.5 µg/mL, and slightly lower activity against *Bacillus subtilis* and *E. coli* (MIC 125 µg/mL) [105]. Verbascoside isolated from *Buddleja globosa* Hope also showed antimicrobial activity against laboratory control strains of *S. aureus* ATCC 25923 and *E. coli* ATCC 25922 with MIC values of 1 mM [162]. The antistaphylococcal activity of verbascoside isolated from *Buddleja cordata* was also observed (MIC of 400 µg/mL), with inhibition of leucine adsorption and blocking of protein synthesis considered a possible mechanism of action [163]. Verbascoside isolated from *Ballota nigra* L. was found to have moderate antibacterial activity with MIC values of 128 μg/mL for *S. aureus* (including a methicillin-resistant strain) and *Proteus mirabilis* [164]. A mixture of the isomeric compounds verbascoside and isoverbascoside, isolated from *Arrabidaea harleyi* A.H. Gentry (Bignoniaceae), showed activity against Gram-positive bacteria (*S. aureus*, *Micrococcus luteus*, *B. subtilis*, *B. mycoides*, *Enterococcus faecalis*), and Gram-negative bacteria (*E. coli*, *Serratia marcensis*), with MIC values in the range of 300~600 μg/mL, but no activity against *P. aeruginosa* and *Mycobacterium smegmatis* was detected [122].

In addition to the antimicrobial activity of verbascoside, synergism with conventional antimicrobial agents with inhibitors of cell wall synthesis and protein synthesis was investigated. When verbascoside and gentamicin were combined, a two-fold reduction in the MIC values of gentamicin against the laboratory control strain of methicillin-resistant *S. aureus* (MRSA) and clinical isolates of *S. aureus* and *E. coli* was observed. However, this combination was only categorized as partial synergy, as most combinations were in the indifferent range [165]. The combination of verbascoside with the inhibitors of cell wall synthesis, vancomycin and ceftazidime, showed a significant reduction of both antibiotics by up to 32-fold, and synergistic effects against multidrug-resistant strains of *S. aureus* and *P. aeruginosa* [154]. These results underline the potential of verbascoside-antibiotic combinations in the treatment of bacterial infections and in the development of antimicrobial resistance.

Extracts from plants rich in verbascoside have higher MIC values compared to isolated verbascoside. Ethanol and chloroform extracts from the aerial part of *Leonurus turkestanicus* V.I. Krecz. et Kuprian. which is rich in verbascoside showed antimicrobial activity against several ATCC reference microorganisms with MIC values in the range of 5~20 mg/mL for Gram-negative bacteria (*E. coli*, *K. pneumoniae*, *P. aeruginosa*, *P. mirabilis*), MIC values of 1.25–20 mg/mL for Gram-positive bacteria (*S. aureus*, *S. epidermidis*, *M. luteus*, *B. subtilis*, *B. cereus*) and MIC 2.5–5 mg/mL for fungi (*Candida albicans*, *C. parapsilosis*) [166]. Similarly, dry hydroethanolic leaf extracts from six Mediterranean olive varieties (Croatian: *Lastovka*, *Levantinka*, *Oblica*; Italian: *Moraiolo*, *Frantoio*, *Nostrana di Brisighella*) were found to have no antimicrobial activity against *E. coli* and *Salmonella* Typhimurium, and showed weak activity against *S. aureus*, *B. cereus*, and *Listeria innocua*, as well as inhibitory activity against *Campylobacter jejuni* at 0.5 mg/mL [167]. In addition, extracts isolated from the plant *Verbena carolina* (Verbenaceae) showed antibacterial effects against *E. faecalis and S. typhi* with MIC values of 1.5 mg/mL [168], and from *Verbascum mucronatum* Lam., with a MIC of 256 μg/mL against *E. faecalis* [112]. Combinations of extracts from verbascoside-rich plants with conventional antibiotics were mostly in the range of indifference, or partial synergy [165].

In addition, the antibacterial activity of verbascoside was also investigated with regard to its possible use in food preservation. A commercial extract of *Lippia citriodora* containing 25% verbascoside showed antimicrobial activity against two pathogenic strains of *E. coli* (O157:H7 and O111) producing verotoxins (VTECs) and two control strains (*E. coli* ATCC 25922 and *E. faecalis* ATCC 29212). The experiments were performed under different storage temperatures (standard room temperature, refrigeration) and pH conditions in food to simulate the environmental conditions associated with VTEC outbreaks. The highest MIC values (7500–10,000 µg/mL) and MBC values (10,000 µg/mL) were detected at 35 °C/pH 5.5, and the lowest at 4 °C/pH 5.5 (MIC ≤ 78.12 µg/mL; MBC 8333 µg/mL), indicating that verbascoside has a strong antimicrobial potential in foods stored under market conditions [169]. Verbascoside has potent antimicrobial activity against common pathogenic multidrug-resistant bacteria *S. aureus* and *P. aeruginosa* isolated from patients with food poisoning, with MIC values in the range of 625–2500 μg/mL [154]. Moderate (2 × MIC) to high (4 × MIC) concentrations of verbascoside sprayed on the surface of meat samples significantly reduced the total number of colony-forming units in chicken, beef, tuna, and pork for nine days, extending the shelf life of various meats [154].

**Figure 4:**
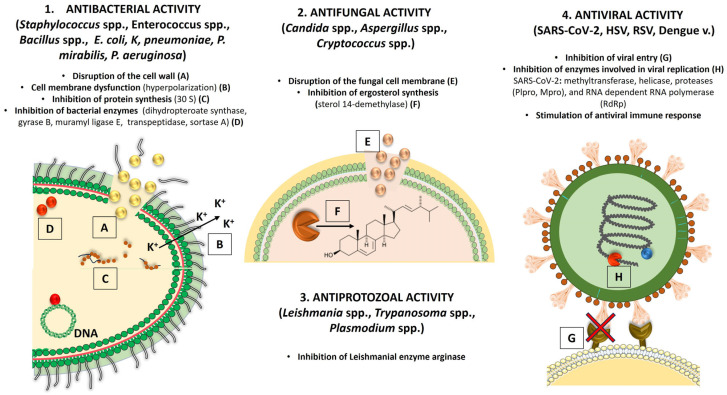
Main mechanisms of antimicrobial activity of verbascoside.

The exact mechanisms of the antibacterial activity of verbascoside have not yet been investigated, but the antimicrobial activity could be the result of several mechanisms, such as disruption of the cell wall, dysfunction of the cell membrane, eradication of the preformed biofilm, inhibition of protein synthesis and alteration of cell morphology (Figure 4) [3,154,163]. Changes in membrane permeability and membrane integrity occur because verbascoside forms channels in the bacterial cell membrane that cause a decrease in ATP concentration, a decrease in cytoplasmic pH, and changes in membrane potential (i.e., hyperpolarization) [154]. The ability of verbascoside to penetrate microbial cell walls and membranes is influenced by its chemical structure. Verbascoside is an amphipathic molecule with hydrophilic (sugar moieties) and hydrophobic (phenol and caffeoyl groups) domains that enable it to interact with both the lipid bilayers of cell membranes and the aqueous environment inside and outside the cell. In addition, the phenolic groups penetrate the lipid bilayers, disrupt the integrity of the membrane, and lead to leakage of cell contents [1].

The effects of verbascoside on the 30 S ribosomal subunit and the bacterial enzymes dihydropteroate synthase, gyrase B, muramyl ligase E, and transpeptidase were investigated in a molecular docking screen of verbascoside isolated from the aerial parts of *Antirrhinum majus* [170]. Verbascoside showed a moderate binding affinity to the 30 S ribosomal subunit of Gram-positive and Gram-negative bacteria (binding energy −5.95 and −6.30 kcal/mol). The binding affinity to bacterial enzymes also differed between Gram-positive and Gram-negative bacteria, not only by the binding energy (dihydropteroate synthase −6.70 and −7.44 kcal/mol, gyrase B −6.05 and −6.47 kcal/mol, muramyl ligase E −5.55 and −6.71 kcal/mol, transpeptidase −7.26 and −6.97 kcal/mol), but also by the different amino acids involved in the formation of hydrogen bonds [170]. Verbascoside also inhibits the enzyme sortase A of *S. aureus*, reducing bacterial adhesion, invasion, and biofilm formation [3].

### 5.2. Antifungal and Antiprotozoal Activity

Medically important fungi and protozoa are of particular interest because they belong to the eukaryotic organisms and their cells are similar in structure to human cells, so that the selective toxicity of antifungal and antiprotozoal agents is lower compared to conventional antibiotics. In addition, treatment is complicated by the limited availability of effective therapies and the emergence of resistant strains, highlighting the urgent need for new antifungal/antiprotozoal agents and strategies. Another major challenge in the treatment of protozoal infections is the complexity of the life cycle of protozoa with different developmental stages in which they reside intracellularly and/or extracellularly in different hosts, which further complicates effective treatment [171,172].

#### 5.2.1. Antifungal Activity

The antifungal activity of verbascoside has been evaluated against *Candida* spp., *Aspergillus* spp., and *Cryptococcus* spp., which are the main causes of fungal infections in immunocompromised patients, e.g., patients with HIV/AIDS, cancer, or organ transplants. These infections can lead to life-threatening conditions such as disseminated candidiasis, pulmonary aspergillosis, and cerebral cryptococcosis, which are associated with a high rate of fatal outcomes [173].

Verbascoside and isoverbascoside isolated from *Pyrostegia venusta* (Ker Gawl.) Miers showed strong anticandidal activity against several laboratory control strains and clinical isolates of *Candida albicans* and the non-albicans species *C. kruzei*, *C. parapsylosis*, *C. tropicalis*, and *guilliermondii* with MIC values in the range of 0.7–1.5 μg/mL for verbascoside and 0.7–6 μg/mL [5]. Moderate anticandidal activities of verbascoside isolated from the floral parts of *Verbascum mucronatum* Lam. were detected against *C. albicans*, *C. kruzei*, and *C. parapsylosis* with MIC values of 256 μg/mL [112]. Similar results were obtained with verbascoside isolated from *Lippia salviaefolia* with MIC values of 125 μg/mL for all three *Candida* species [174]. The strongest activity of *L. salviaefolia* verbascoside was found against *Cryptococcus neoformans* with an MIC of 15.6 μg/mL, which was almost four times higher compared to amphotericin B (AmB) (MIC of 4.0 μg/mL) [174].

Several studies investigated the fungicidal and biofilm-eradicating effect of verbascoside in combination with AmB against reference strains and clinical isolates of *Candida* spp., *Aspergillus* spp., and *Cryptococcus* spp. [158,175]. Verbascoside isolated from the aerial parts of *Colebrookea oppositifolia* exhibited moderate antifungal activity against all tested fungal strains, with MIC values above 12.5 μg/mL. Synergistic fungicidal activity and prolonged postantifungal activity were observed with the combination of 3.12 and 12.5 µg/mL verbascoside with sub-inhibitory concentrations of AmB. This combination also reduced the minimum biofilm reduction concentrations of AmB by 2-16-fold for *Candida albicans*, *Cryptococcus neoformans*, and *Aspergillus fumigatus*. The authors suggested that the synergistic effect is due to the facilitated uptake of verbascoside triggered by subinhibitory concentrations of AmB, which enhances the fungicidal effect [175]. Since pure verbascoside has very low oral bioavailability, modification with enzymes may improve its pharmacological and pharmacokinetic properties. Khazir et al. investigated the effect of selective acylation of verbascoside with lipase B from *Candida antarctica*, resulting in verbascoside analogues such as verbascoside 4″-octanoate, verbascoside 4″-palmitate, and verbascoside 4″,4′-palmitate. These analogues showed a superior synergistic effect with subinhibitory concentrations of AmB, and reduced its MIC by up to fourfold, compared to verbascoside [158].

Besides the above-mentioned fungal species, extracts isolated from plants rich in verbascoside, such as *Verbena carolina* (Verbenaceae), *Buddleja thyrsoides* Lam, *Lippia javanica*, and *Lantana camara*, showed antifungal effects against the dermatophytes *Trichophyton mentagrophytes* and *T. rubrum* [168], *Saccharomyces cerevisiae* [176], *and Penicillium digitatum* [177].

Although the literature on the antifungal activity of verbascoside is scarce, possible mechanisms of action include disruption of the fungal cell membrane, leading to cell lysis and death, and inhibition of ergosterol synthesis, which is the essential component of fungal cell membranes (Figure 4) [3]. A molecular docking screen of verbascoside isolated from the aerial parts of *Antirrhinum majus* showed a high binding affinity to *C. albicans* sterol 14-demethylase (binding energy −9.40 kcal/mol), forming two hydrogen bonds with the amino acids HIS468 and MET508 of the enzyme. This effect was comparable to the standard antifungal agent fluconazole, which inhibits sterol 14-demethylase with a binding energy of −7.12 kcal/mol and four hydrogen bonds with the amino acids HIS377, SER378, PHE380, and MET508 [170].

#### 5.2.2. Antiprotozoal Activity

The antiprotozoal activity of verbascoside was mainly investigated in vitro against *Leishmania* spp. and *Trypanosoma* spp. Leishmaniasis infections are of major public health importance worldwide and affect millions of people in tropical and subtropical regions. These infections can occur in several forms, including cutaneous, mucocutaneous, and visceral leishmaniasis, each with its severe health consequences. Visceral leishmaniasis, also known as kala-azar, is particularly deadly if left untreated and leads to severe organ damage and a high mortality rate [178]. Similar to leishmaniasis, trypanosome infections are important due to their serious health effects, including African sleeping sickness and Chagas disease, which can lead to neurological damage, cardiac complications, and death if left untreated [179]. The complexity of the life cycle of both protozoa and the emergence of drug-resistant strains underscores the urgent need for effective antiprotozoal treatments and the importance of ongoing research in this field.

Several studies investigated the antileishmanial activity of verbascoside against different *Leishmania* species and different stages of the life cycle (amastigote and promastigote), as well as the inhibitory effect on the enzyme arginase, which is crucial for the replication and infectivity of the parasite [6,180,181]. Previous studies have shown that the absence of arginase activity leads to reduced infectivity of *Leishmania amazonensis* [182]. Verbascoside exhibits antileishmanial activity against *Leishmania infantum*, *L. donovani*, and *L. amazonensis*. It showed an effective concentration value (EC_50_) of 19 μM against extracellular promastigotes of *L. amazonensis* [6] and EC_50_ of 32μM against intracellular amastigotes of *L. amazonensis* [181]. At both stages of the life cycle, verbascoside acted as a competitive arginase inhibitor, and the interaction with arginase was confirmed by docking studies, indicating the potential of verbascoside for the development of new treatments for leishmaniasis.

Verbascoside isolated from *Phlomis brunneogaleata* Hub.-Mor. (Lamiaceae) showed pronounced antileishmanial activity against axenic *L. donovani* amastigotes with inhibitory concentration values (IC_50_) of 8.7 μg/mL. Isoverbascoside showed similar efficacy with an IC_50_ of 9.2 μg/mL. In addition, both compounds were found to have good antiprotozoal activity against trypomastigote forms of *Trypanosoma b. rhodesiense* (verbascoside IC_50_ 14.2 μg/mL, isoverbascoside IC_50_ 6.2 μg/mL), while the inhibitory concentration for trypomastigote forms of *T. cruzy* was above 90 μg/mL for both compounds and for *Plasmodium falciparum* above 50 μg/mL for verbascoside and 37.5 μg/mL for isoverbascoside [183]. Similar results were reported by the same authors when the in vitro antiprotozoal activity of *Ajuga laxmannii* (Lamiaceae) and its secondary metabolites were investigated [184]. In addition, verbascoside was one of the major components among phenylethanoid glycosides isolated from a traditional antimalarial medicinal plant, *Stachytarpheta cayennensis* (Rich.) Vahl, Verbenaceae, suggesting a potential antimalarial activity of this compound [185]. On the other hand, verbascoside isolated from the leaves of *Clerodendrum chinense* (Lamiaceae) from Egypt showed marginal activity against *T. cruzi* with an IC_50_ of 32.81 μM, and other protozoa above 64 μM [186].

### 5.3. Antiviral Activity

Medicinal plants have shown considerable potential for the treatment of viral infections as they contain a variety of bioactive compounds with different mechanisms of action that act synergistically on different phases of viral replication [187,188]. These plants and their compounds represent a promising alternative to the limited antiviral therapies and address the growing concern about resistance to antiviral agents [189]. Among them, verbascoside has shown notable antiviral activity, not only through a direct antiviral action but also by modulating the host immune response. Several in silico and in vitro studies have demonstrated strong inhibitory effects of verbascoside against a number of viruses, including *Coronaviridae* (SARS-CoV-2, HCov-229E), *Herpesviridae* (herpes simplex virus 1 and 2 (HSV-1 and HSV-2), Aujeszky virus-Suid herpesvirus 1 (SuHV-1)), *Flaviviridae* (Dengue virus), *Pneumovoridae* (respiratory syncytial virus (RSV)), HBV, influenza virus, and enteroviruses [35,190,191,192]. Antiviral effects are achieved by inhibiting the attachment and penetration of viruses into the host cells and by inhibiting viral enzymes and replication (Figure 4).

SARS-CoV-2 is the cause of the severe respiratory disease COVID-19, and its complications, including pneumonia, acute respiratory distress syndrome (ARDS), and multi-organ failure [193]. A recent in silico-docking study has shown that verbascoside isolated from olive leaves has a strong inhibitory effect on viral enzymes of SARS-CoV-2, that are essential for viral replication and fitness. Verbascoside inhibited the SARS-CoV-2 methyltransferase, helicase, proteases (the papin-like protease Pl^pro^ and the main protease M^pro^), and the RNA-dependent RNA polymerase (RdRp) with high docking scores [191,194,195]. In addition, an in vitro antiviral assay on Vero-E6 cells infected with hCoV-19/Egypt/NRC-03/2020 confirmed a moderate antiviral activity of the standardized olive leaf extract containing 20% oleuropein against SARS-CoV-2 with an IC_50_ of 118.3 μg /mL [191]. Although only a few studies have investigated the therapeutic use of verbascoside in the treatment of COVID-19, the results of in silico and in vitro studies suggest a promising antiviral potential of verbascoside against SARS-CoV-2 [3].

HSV-1 and HSV-2 primarily cause oral and genital herpes, respectively, and can lead to severe complications such as encephalitis, neonatal herpes, ocular infection, and increased susceptibility to HIV infection in immunocompromised patients [196]. Verbascoside isolated from *Lepechinia speciosa* has shown the ability to inhibit the in vitro replication of HSV-1 and HSV-2 on Vero cells. The mechanisms of action include both direct virucidal activity and inhibition of viral entry into host cells. Verbascoside exhibits dose-dependent antiviral activity with an EC_50_ of 58 μg/mL for HSV-1 and 8.9 μg/mL for HSV-2. The virucidal activity against HSV-1 is achieved by interacting with viral particles and preventing their adsorption, with an inactivation of 82.2% and a similar inhibition during the intracellular phase. In HSV-2, verbascoside interacts with cellular receptors and prevents 92% of the virus attachment and penetration [35].

RSV infections are of great importance as they can cause severe respiratory disease, particularly in infants, young children, and the elderly, leading to complications such as bronchiolitis, pneumonia, and in some cases hospitalization and death [197]. The antiviral activity of *Plantago asiatica* and *Clerodendrum trichotomum* extracts and their main component, verbascoside, against RSV has been studied in vitro, on human epithelial type *2* cells (HEp-2) and A549 cells, and in vivo on the mouse model of RSV infection [198]. Pure verbascoside significantly inhibited RSV replication with an EC_50_ of 15.64 ± 1.07 ng/mL and a cytotoxic concentration (CC_50_) of verbascoside of 740.34 ± 8.23 ng/mL, reduced viral titers determined by the plaque formation assay, reduced HEp-2 cell death induced by RSV infection, and reduced mRNA and protein expression of viral genes in verbascoside-treated HEp-2 cells. The level of viral mRNA in the lungs of RSV-intranasally infected BALB/c mice was significantly lower when treated with 80 mg/kg verbascoside. The results of this study indicate that the tested herbal extracts and verbascoside have a strong antiviral effect both in vitro and in vivo and inhibit the main factors contributing to viral pathogenicity [198].

The effect of verbascoside on IFN-γ production in vitro and in vivo (in C57BL/6 and Balb/c mice) following infection with mouse-adapted influenza virus (A/FM/1/47 H1N1, FM1) or the NJ strain of vesicular stomatitis virus (VSV) showed that verbascoside effectively stimulates IFN-γ secretion in T cells at transcriptional and translational levels. Since the secretion of this cytokine plays a central role in immunity against viruses, this represents one of the mechanisms of the antiviral effect of verbascoside [156].

The antiviral activity of verbascoside was also investigated against Dengue virus-2 in Vero and LLCMK2 cells treated with verbascoside for 48 h, with an EC_50_ of 3.4 ± 0.4 μg/mL [199]. However, the mechanisms of the antiviral effect have not yet been clarified. In addition to the studies in which antiviral effects of pure verbascoside were observed, plant extracts from the leaves and flowers of *Verbascum thapsus* L., which are rich in verbascoside, exhibited antiviral activities against HCov-229E, HBV and HSV-2 [192] and the ethanolic extracts from leaves and stems of *Arrabidaea samydoides* (Cham.) Sandw. which are rich in verbascoside, have shown antiviral activities against HHV-1, encephalomyocarditis virus (EMCV), and vaccinia virus (VACV) [199].

Antimicrobial activity of verbascoside is summarized in Supplementary Table S2.

## 6. Anti-Inflammatory and Immunomodulatory Effects of Verbacoside

The biological effects of verbascoside, such as anti-inflammatory, immunomodulatory and antioxidant, and the underlying mechanisms have been confirmed by in vitro and in vivo studies.

The mechanism of the anti-inflammatory effect is not fully understood, but the ability of verbascoside to inhibit the release of arachidonic acid (AA) and histamine may be involved in its anti-inflammatory action, based on results obtained on RBL-2H3 mast cells [200,201] where it competitively inhibited Ca^2+^-dependent phospholipase A(2) (cPLA(2)) [201]. It has also been suggested that the anti-inflammatory effect of verbascoside may be related to its inhibitory effects on inducible nitric oxide synthase (iNOS) and NO production. Pesce et al. reported that verbascoside increases the activity of Src homology region 2 domain-containing phosphatase-1 (SHP-1), which downregulates TAK-1/JNK/AP-1 signaling and inhibits the expression and activity of cyclooxygenases 2 (COX2) and iNOS in LPS-stimulated U937 human mononuclear cells [202]. Also, verbascoside has been reported to inhibit the expression of iNOS and/or NO production in LPS-stimulated murine RAW 264.7 [203,204,205] and J774.A1. macrophage cell lines [206,207], mouse peritoneal macrophages [208,209], human monocyte THP-1 cell line [210], and rat glioma C6 cell line [211]. In LPS-stimulated RAW 264.7 cells, in addition to inhibiting iNOS/NO production, verbacoside, through activation of p38 and Nrf2 expression, induces heme oxygenase-1 (HO-1) which in turn reduces high mobility group box 1 (HMGB1) release in both macrophages and cecal ligation and puncture (CLP)-induced mouse sepsis model [203]. Moreover, in LPS-stimulated mouse peritoneal macrophages, verbascoside inhibited production of TNF-α [208,209] and IL-12 [209], whereas in LPS-stimulated human umbilical vein endothelial cells (HUVEC), this compound inhibited production of TNF-α and IL-1β [104]. In LPS-stimulated monocyte THP-1 cells, verbascoside inhibited the expression of thymic stromal lymphopoietin (TSLP), IL-1β, TNF-α, and IL-8 [210]. Also, another study reported that verbascoside downregulated expression and activity of iNOS, O_2_^−^ formation, and superoxide dismutase (SOD), catalase and glutathione peroxidase activity in THP-1 cells stimulated with LPS and IFN-γ implying that its anti-inflammatory properties come, at least partly, from its ability to reduce the production of superoxide radicals and consequently the activity of iNOS [212].

The inhibitory effect of verbascoside on the production of pro-inflammatory cytokines (IL-6, IL-12, TNF-α, and IFN-γ) via inactivation of the JAK/STAT signaling pathway was also reported in IL-1β-stimulated primary rat chondrocytes [213], implying its therapeutic potential in osteoarthritis (OA). The findings were confirmed in vivo in the rat model of OA [213]. Chang et al. demonstrated that verbascoside decreased production of pro-inflammatory cytokines (TNF-α and IL-12) and increased production of anti-inflammatory IL-10 in LPS-stimulated mouse bone marrow-derived dendritic cells (DCs) in vitro. They also showed expansion of Foxp3+ T regulatory (Treg) cells in the coculture of verbascoside-treated DCs with CD4+ T cells. The effects of verbascoside on the activation and function of DCs were mediated through activation of aryl hydrocarbon receptor (AhR) [214].

The effect of verbascoside was investigated on HaCaT cells (immortalized human keratinocytes), which are exposed to a UV-C light source in order to induce necrosis [215]. In this model, verbascoside exerted a protective effect by downregulating the expression of the chemokines CXCL10/IP-10 and CXCL8/IL-8 via suppressing NF-kB and AP-1 binding activity [215]. Furthermore, in A549 cells (human adenocarcinomic alveolar basal epithelial cells) stimulated with TNF-α to establish a model of acute lung injury in vitro, verbascoside decreased expression of IL-1β, IL-8, IL-6, and caspase-3, -8, and -9. Also, antioxidant factors HO-1, glutamate cysteine ligase (GCLC), and NAD(P)H quinone oxidoreductase 1 (NQO1) were upregulated [216]. The suggested underlying mechanisms for these effects are the upregulated expression of Keap1, the enhanced activation of Nrf2, and the decreased expression of p-IκBα and nuclear p65 [216]. In the same cell line A549, but when using LPS as a stimulator, Jing et al. found that verbascoside inhibited the NF-kB signaling pathway by inhibiting the phosphorylation of IκBα, NF-κBp65, IKK-α, and IKKβ [217].

Since mast cells have a role in allergic and other mast-cell-mediated inflammatory diseases, as well as in tumour growth [218], Yoou et al. explored the effects of verbascoside on TSLP-stimulated human mast cell line (HMC-1) for its potential therapeutic use [219]. This study showed that verbascoside downregulated murine double minute 2 (MDM2), a protein involved in mast cell proliferation and a negative regulator of p53. It also reduced the production of IL-13, IL-6, TNF-α, and IL-1β and induced the activation of caspase-3 [219]. The same study reported the cleavage of poly-ADP-ribose polymerase, reduction of the procaspase-3 and Bcl2, and inhibition of expression of TSLP receptor and IL-7R [219].

There are several findings that support the anti-allergic potential of verbascoside. The verbascoside inhibits β-hexosaminidase release and decreases intracellular Ca^2+^ level in rat leukemia basophilic cells (RBL-2H3) sensitized with IgE and inhibits histamine release and TNF-α and IL-4 production in stimulated human basophilic KU812 cells [220]. Another study, performed also on the stimulated KU812 cells, reported that verbascoside downregulates the expression of CCL1-4, FCER1A, and NFATC1 genes, decreases JNK phosphorylation, and inhibits the MAPK signaling pathway [221]. Since IL-32 plays a role in the pathogenesis of different chronic inflammatory diseases [222,223] as well as in the development of allergic rhinitis [224], for exploring verbascoside effects on allergic inflammatory responses, Nam et al. used IL-32-stimulated monocyte THP-1 cells as in vitro model for macrophage—mediated allergic inflammation [210]. They reported that verbascoside inhibited the expression of TSLP, IL-1β, TNF-α, IL-8, NO, and iNOS, and inhibited caspase-1 activation in IL-32 stimulated THP-1 cells, suppressed nuclear translocation and binding activities of NF-kB, and reduced phosphorylation of IƘB-α [210]. Moreover, in an in vitro model of atopic dermatitis in which THP-1 cells were incubated with the contact allergen 2,4-dinitrochlorobenzene (DNCB), verbascoside suppressed the expression of the costimulatory molecules CD86 and CD54, which are important for T cell activation, and reduced production of pro-inflammatory TNF-α and IL-6 cytokines [225]. The proposed mechanism underlying these effects was downregulation of NF-kB signaling [225].

In vitro studies exploring the effects of verbascoside were mainly conducted on cells that represent cells of innate immunity, showing its anti-inflammatory and anti-oxidative effects. However, the study of Wu et al. was performed on cells of adaptive immunity—B cells, implying an immunomodulatory effect of this compound. This group reported that verbascoside promoted the production of IL-10 by human and murine B cells after stimulation with LPS, i.e., upon engagement of TLR4, and that the TLR4/PI3K axis signaling is a critical target for this compound [226]. A summary is presented in Table 1.

The anti-inflammatory and immunomodulatory effects of verbascoside were also examined in various animal models of inflammatory and immune-mediated diseases.

In carrageenan-induced rat paw edema, verbascoside inhibited edema formation, demonstrating its anti-inflammatory effect in this classical model of acute, non-immune inflammation [227]. In another model of acute inflammation in mice, LPS-induced acute lung injury, verbascoside reduced the lung wet-to-dry weight ratio, myeloperoxidase (MPO) activity, and histopathological lung damage. Additionally, it increased the antioxidant parameter SOD and decreased malondialdehyde (MDA) levels, inflammatory cell infiltration and pro-inflammatory cytokines (TNF-α, IL-1β, IL-6) in bronchoalveolar lavage fluid [217]. In a model of CLP-induced sepsis, the “gold standard” rodent model for abdominal sepsis [228], verbascoside improved survival rates and reduced HMGB1 levels in serum and lung tissue of treated mice, indicating that it may be useful in the treatment of sepsis [203]. Furthermore, verbascoside reduced pro-inflammatory cytokines (IL-1β, IL-6, IL-12, TNF-α, IFN-γ) and apoptosis markers (Bax, cleaved caspase-3) in the rat OA model, while it enhanced anti-apoptotic Bcl2 expression in cartilage [213].

Verbascoside has also been reported to exhibit a protective effect in models of chronic intestinal inflammation. In 2,4-dinitrobenzene sulfonic acid (DNBS)-induced colitis, treatment with verbascoside improved macroscopic damage, body weight, and inflammatory markers (TNF-α, IL-1β) while reducing oxidative stress and metalloproteinase activity (MMP-2 and MMP-9) in colon tissue [229,230]. It was reported that PPAR-α can contribute to the anti-inflammatory activity of verbascoside according to the results obtained in this experimental model performed on PPAR-αKO and PPAR-αWT mice [230]. In dextran sodium sulfate (DSS)-induced colitis in C57BL/6 mice, verbascoside alleviated disease symptoms, modulated cytokine expression (upregulated IL-10, downregulated IL-1β and TNF-α), and inhibited JAK2/STAT3 and NF-κB pathways. It also reduced oxidative stress by decreasing the production of pro-oxidants MDA and NO, and increasing the production of anti-oxidants glutathion (GSH), SOD, and Nrf2 and HO-1 levels in colon tissue [85,231]. Guo et al. pointed out that verascoside achieved a favorable effect on colon inflammation through downregulation of protein expression of HMGB1, a central player in the initiation and progression of ulcerative colitis, and upregulation of HO-1 levels in colon tissues [231].

In an ovalbumin (OVA)-induced asthma model, verbascoside reduced Th2 cytokines (IL-4, IL-5, IL-13) and OVA-specific IgE antibody production, and promoted Treg cell differentiation, reducing airway hyperresponsiveness and the accumulation of inflammatory cells in the lungs [214].

Furthermore, in a model of 2,4-dinitrochlorobenzene (DNCB)-induced atopic dermatitis in mice, topical application of verbascoside reduced scratching and skin lesion severity, lowered IgE and IL-4 and IL-13 levels, and decreased inflammatory cytokines (TNF-α, IL-6, IL-4) in affected skin [225].

The anti-inflammatory/immunomodulatory effect of verbascoside has also been confirmed in animal models of autoimmune diseases. In an animal model of Sjögren syndrom, verbascoside improved salivary flow, reduced autoantibody levels, and modulated the frequency and activity of T and B cell populations (decreased the frequency of effector CD4+IFN-γ+Th1, CD4+IL-17+Th17 and CD4+PD-1+ICOS+Tfh cells and enhanced the IL-10 producing capacity of splenic B regulatory cells and TLR4+CXCR4+plasma cells) [226]. In experimental autoimmune encephalomyelitis (EAE), an animal model of multiple sclerosis, verbascoside delayed disease progression and reduced inflammatory infiltration, demyelination, and oxidative stress in the spinal cord. It also suppressed peripheral immune activation by reducing pro-inflammatory cytokines and chemokines [232]. (A summary is presented in Table 2).

**Figure 5:**
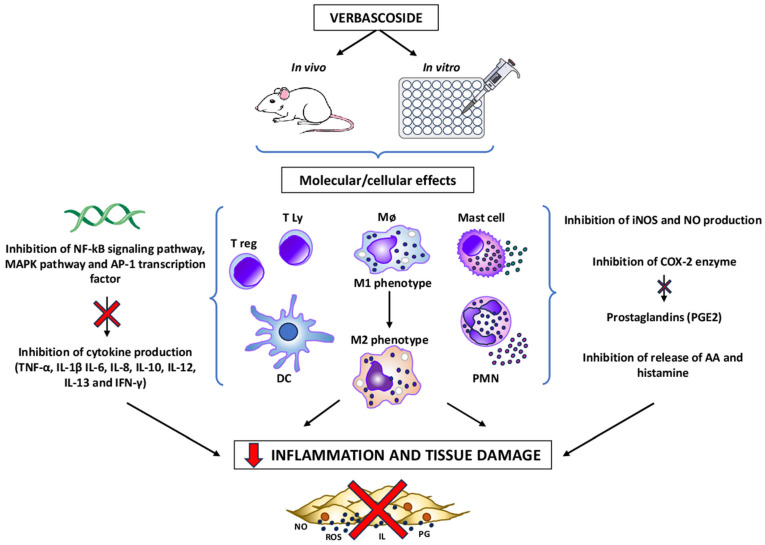
In vivo and in vitro anti-inflammatory and immunomodulatory effects of verbascoside.

In vivo and in vitro anti-inflammatory and immunomodulatory effects of verbascoside are summarized in Figure 5.

The effects of verbascoside are expressed in T lymphocytes (T ly), macrophages (Mø), regulatory T lymphocytes (T reg), dendritic cells (DC), mast cells, and polymorphonuclear leukocytes (PMN). Verbascoside promotes the shift from the M1 (pro-inflammatory) to the M2 (anti-inflammatory) phenotype of Mø, balances Th1/Th2 responses, promotes Treg proliferation, and reduces degranulation of mast cells and PMN, as well as the production and release of reactive oxygen species (ROS).

Molecular targets of verbascoside in these cells are the NF-κB and MAPK signaling pathway, and the transcription factor AP-1, leading to a significant change in the production of the pro- and anti-inflammatory cytokines TNF-α, IL-1β, IL-6, IL-8, IL-10, IL-12, IL-13, and IFN-γ, both in vivo, in animal models of inflammatory diseases, and in vitro. Verbascoside also inhibits inducible nitric oxide synthase (iNOS) and cyclooxygenase-2 (COX-2) and reduces the production of nitric oxide (NO), ROS, and prostaglandins (PGE2), as well as the release of arachidonic acid (AA) and histamine. Molecular and cellular mechanisms by which verbascoside exerts its action on the components of the immune system lead to a decrease in inflammation and an attenuation of tissue damage in models of colitis, arthritis, and neuroinflammation.

## 7. Conclusions

In summary, the in vitro studies indicate that verbascoside has minimal cytotoxic effects on most cell lines at low concentrations. Selective cytotoxicity was observed in several cancer cell lines, whereas normal human cells showed no signs of cytotoxicity. Verbascoside even promoted the growth and proliferation of normal lymphocytes and normal human embryonic diploid lung fibroblasts and delayed senescence. In vivo studies confirmed no acute or subacute toxicity, confirming the safety and therapeutic use of verbascoside.

The antistaphylococcal activity of verbascoside is based on the inhibition of leucine adsorption and the blocking of protein synthesis. It also inhibits sortase A in *Staphylococcus aureus* and thus reduces bacterial adhesion, invasion, and biofilm formation. Other antibacterial mechanisms include disruption of the cell wall, membrane dysfunction, eradication of biofilms, inhibition of protein synthesis, and alteration of cell morphology.

The antifungal mechanisms include the disruption of the fungal cell membrane, which leads to lysis and death, and the inhibition of ergosterol synthesis by targeting sterol-14 demethylase. The antileishmanial effect of verbascoside is based on the inhibition of arginase in *Leishmania* amastigotes and promastigotes, which is crucial for the replication and infectivity of the parasite.

Verbascoside’s main antiviral mechanisms include the inhibition of viral enzymes that are important for replication and the stimulation of the immune response against viruses.

Verbascoside demonstrated potent anti-inflammatory, antioxidant, and immunomodulatory effects across diverse in vitro and in vivo animal models of inflammation and immune-mediated diseases. These properties highlight its potential therapeutic applications, or at least its beneficial effects in both inflammatory and autoimmune conditions.

## Figures and Tables

**Table 1:** Anti-inflammatory/immunomodulatory effects of verbascoside in different in vitro models.

The Source of Verbascoside (Plant Extract/Compound)	Tested Concentrations	Biological Effect	Cell Line/Cells	Refs.
*Clerodendron trichotomum*	1–10 µM	Inhibition of release of AA and histamine, and PGE2 production; competitive inhibition of cytosolic Ca^2+^-dependent PLA2	RBL-2H3	[200,201]
Verbascoside	10 μg/mL	Inhibition of NO production	RAW 264.7	[204]
*Wendita calysina*	0.05 mg/mL, 0.1 mg/mL and 0.5 mg/mL	Inhibition of NO production	J774.A1	[206]
*Buddleja officinalis*	10 µM, 30 µM, 100 µM	Inhibition of iNOS gene expression; suppression of NF-kB and AP-1 activation	RAW 264.7	[205]
*Verbascum thapsus*	50 µM	Downregulation of expression and activity of COX2 and iNOS; downregulation of TAK-1, JNK, AP-1 activation via activation SHP-1	U937	[202]
Verbascoside	1 µM, 10 µM, 100 µM	Inhibition of NF-KB pathway	A549	[217]
*Scrophularia scorodonia*	25 µM, 50 µM, 100 µM	Inhibition of NO, PGE2, and TNF-α production	Mouse peritoneal macrophages	[208]
*Buddleja officinalis*	1 µmol/L, 10 µmol/L, 100 µmol/L	Inhibition of NO, TNF-α, and IL-1β production	HUVEC	[104]
*Verbascum thapsus*	100 µM	Downregulation of expression and activity of iNOS, O_2_^−^ formation, and SOD, CAT and GPx activity; diminished expression of NF-kB	THP-1	[212]
*Anisomeles indica*	40 µM	Inhibition of NO, IL-12, and TNF-α production	Mouse peritoneal macrophages	[209]
*Kigelia africana*	0.1–1 mM	Inhibition of NO production	J774.A1	[207]
Verbascoside	10 µM, 50 µM, 100 µM	Inhibition of HMBG1 release, expression of iNOS and production of NO; increase of HO-1 expression; induction of p38 MAPK/Nrf2 signal pathways	RAW 264.7	[203]
Verbascoside	10 µM, 50 µM, 100 µM	Inhibition of IL-6, IL-12, TNF-α, and IFN-γ production; enhanced viability; inhibition of JAK/STAT signaling pathway; enhanced Bcl2 expression and dampened Bax/cleaved-caspase 3 expression	IL-1beta stimulated primary rat chondrocytes	[213]
*Syringa vulgaris* vegetal cells	0.1 µM, 1 µM, 10 µM, 50 µM	Inhibition of CXCl10/ IP-10 and CXCL8/IL-8 production; impaired NF-κB and AP-1 DNA binding activity	HaCaT	[215]
*Syringa vulgaris* IRBSV25/B cells	10 μg/mL, 50 μg/mL, 100 μg/ml	Inhibition of iNOS expression and NO production, inhibition of COX2 expression; inhibition of the activation of NF-kB and ERK MAPK signaling pathway	C6	[211]
*Callicarpa kwangtungensis*	10 μM, 20 μM, 40 μM	Inhibited apoptosis; decreased expression of IL-1β, IL-8, IL-6; decreased levels of caspase -3, -8, -9; upregulated HO-1, GCLC, and NQO1; upregulated expression of Keap1, enhanced activation of Nrf2, decreased expression of p-IκBα and nuclear p65	TNF-α stimulated A549	[216]
*Radix Rehmanniae*	25 μM, 50 μM	Promoted production of IL-10; enhanced PI3K/Akt signaling	LPS-stimulated human and murine B cells	[226]
*Cistanche deserticola*	1 μM, 10 μM, 50 μM	Increased production of IL-10; decreased production of IL-12, and TNF-α; enhanced expansion of Foxp3 Tregs; AhR activation	LPS-stimulated mouse BMDC	[214]
*Cistanche tubulosa*	0.1 μg/mL, 1.0 μg/mL, 10.0 μg/mL	Inhibited β-hexosaminidase release and decreased intracellular Ca^2+^ level in RBL-2H3 cells; inhibited histamine release and TNF-α and IL-4 production in KU812 cells	A23187 plus PMA- or 48/80-stimulated KU812 and IgE-sensitized RBL-2H3 cells	[220]
*Cistanche tubulosa*	0.1 μg/mL, 1.0 μg/mL	Downregulated expression of CCL1-4, FCER1A, and NFATC1 genes; decreased JNK phosphorylation and inhibition of the MAPK pathway	A23187 plus PMA- PMA-stimulated KU812	[221]
Verbascoside	0.1 μg/mL, 1.0 μg/mL	Inhibited expression of TSLP, IL-1β, TNF-α, and IL-8, and expression of NO and iNOS, and decreased caspase-1 activation in THP-1 cells; inhibited production of TNF-α in peritoneal macrophages; suppressed nuclear translocation and binding activities of NF-kB and reduced phosphorylation of IƘB-α	IL-32- or LPS-stimulated THP-1 cells and mouse peritoneal macrophages	[210]
Verbascoside	0.1 μg/mL, 1.0 μg/mL	Downregulation of MDM2 and upregulation of p53; reduced production of IL-13, IL-6, TNF-α, and IL-1β; induced activation of caspase-3, the cleavage of poly-ADP-ribose polymerase, and reduction of the procaspase-3 and Bcl2; inhibited expression of TSLP receptor and IL-7 R; increased phosphorylation of STAT 5 and 6	TSLP-stimulated HMC-1 cells	[219]
Verbascoside	5 µM, 10 µM	Suppressed expression of CD86 and CD54; reduced production of TNF-α and IL-6; downregulated activation (phosphorylation) of p65 and IκBα	DNCB-stimulated THP-1	[225]

Abbreviations: AA—arachidonic acid; AhR—aryl hydrocarbon receptor; AP-1—activator protein 1; BMDC—bone marrow dendritic cell; CAT—catalase; COX2—cyclooxygenase 2; DNCB—2,4-dinitrochlorobenzene; GCLC—glutamate cysteine ligase catalytic subunit; GPx—glutathione peroxidase; HMBG1—High Mobility Group Box 1; HO-1—heme oxygenase; iNOS—inducible nitric-oxide synthase; JNK—c-Jun-NH (2)-terminal kinase; LPS—lipopolysaccharide; MAPK—mitogen-activated protein kinase; MDM2—murine double minute 2; NF-kB—nuclear factor kappa-light-chain-enhancer of activated B cell; Nrf2—nuclear factor erythroid 2-related factor 2; NQO1—NADPH quinone oxidoreductase; PGE2—prostaglandin E2; p-IκBα—phosphor IκBα; PMA—phorbol 12-myristate 13-acetate; PLA2—phospholipase A2; SHP-1—Src homology region 2 domain-containing phosphatase-1; STAT—signal transducer and activator of transcription; TAK-1—transforming growth factor-β activated kinase; TSLP—human thymic stromal lymphopoietin.

**Table 2:** Anti-inflammatory/immunomodulatory effects of verbascoside in different in vivo animal models.

The Source of Verbascoside (Plant Extract/Compound)	Tested Doses	Biological Effect	Animal Model	Ref
Verbascoside	30 mg/kg, 60 mg/kg; i.p.	Decreased lung wet-to-dry weight ratio and lung MPO activity; ameliorated histopathological changes; increased SOD level; inhibited MDA content, total cell and neutrophil infiltrations, and levels of pro-inflammatory cytokines (TNF-α, IL-1β, IL-6) in BALF	LPS-induced acute lung injury in BALB/c mice	[217]
*Stachytarpheta cayennensis*	100 mg/kg, 150 mg/kg; p.o.	Inhibition of edema formation	Carrageenan-induced rat paw edema	[227]
Verbascoside	100 mg/kg; i.p.	Increased survival; decreased levels of HMBG1 in serum and lung	CLP-induced sepsis in BALB/c mice	[203]
Verbascoside	100 mg/kg; i.p.	Reduced production of IL-1β, IL-6, IL-12, TNF-α and IFN-γ in synovial fluid; enhanced Bcl2 expression and decreased Bax and cleaved caspase 3 expression; inhibition of JAK/STAT signaling	OA surgery model in SD rats	[213]
*Syringa vulgaris* IRBSV25/B cell cultures	0.2 mg/kg, 2 mg/kg; p.o.	Reduced macroscopic damage score, reduced weight loss, MPO activity, thiobarbituric acid-reactant substances, expression of TNF-α, Il-1β, ICAM-1, P-selectin, iNOS, poly(ADP ribose), NF-κB p65 nuclear levels, and activity of pro-active form of MMP 2 and MMP-9 activity	DNBS-induced colitis in SD rats	[229]
*Syringa vulgaris* IRBSV25/B cell cultures	2 mg/kg; p.o.	Reduces the microscopic and macroscopic signs; inhibited neutrophil infiltration, intestinal permeability, and colon injury more significantly in PPAR-αWT mice	DNBS-induced colitis in PPAR-αKO mice	[230]
*Acanthus ilicifolius* var. *xiamenensis*	100 mg/kg, 200 mg/kg; p.o.	Reduced weight loss and DAI score, suppressed colon shortening, alleviated colon pathological injury; up-regulated IL-10, down-regulated IL-1β and TNF-α; decreased MDA and NO, and increased GSH, SOD, and Nrf2 and HO-1 protein in colon; activated Nrf2, and inhibited protein expression of JAK2/STAT3, NF-κB p65, IKKα/β, and IKB in colon	DSS-induced colitis in C57BL/6 mice	[85]
Verbascoside	30 mg/kg, 60 mg/kg; i.p.	Reversal in body weight loss, colon shortening, DAI score, inflammation, oxidative stress, and colonic barrier dysfunction; inhibited apoptosis in the colon; down-regulated protein expression of HMGB1, and up-regulated HO-1 in colon	DSS-induced colitis in C57BL/6 mice data	[231]
*Radix Rehmanniae*	10 mk/kg; p.o.	Improved salivary flow rate; reduced serum levels of anti- SSA and anti- M3 muscarinic receptor IgG antibodies; reduced IL-17 and increased IL-10 in serum; increased effector T cells (Th1, Th17, Tfh) in LNs, promoted production of IL-10 from B regulatory cells and TLR4+CXCR4+ plasma cells in spleen	Experimental Sjogren’s syndrome in C57BL/6 N	[226]
*Cistanche deserticola*	25 mg/kg, 50 mg/kg; p.o.	Reduced levels of IL-4, IL-5, IL-13; increased IL-10 and TGF-β; promoted CD4+CD25+Foxp3+ Treg differentiation; suppressed specific T cell proliferation; reduced OVA-specific IgE production; attenuated accumulation of inflammatory cells in lungs and development of airway hyperresponsiveness	OVA-induced allergic asthma in mice data	[214]
Verbascoside	1%, topical application	Relieved AD-like symptoms (scratching and skin lesion severity), reduced the levels of total IgE and DNCB-specific IgE, and IL-4 and IL-13 in serum; decreased TNF-α, IL-6, and IL-4 in skin lesions	DNCB-induced AD model in BALB/c mice	[225]
*Radix Rehmanniae*	prevention: 30 mg/kg treatment: 5 mg/kg, 10 mg/kg, 30 mg/kg; p.o.	Attenuated disease severity and progress as prophylactic or therapeutic; in SC: reduced inflammatory infiltration and demyelination; in spleen: reduced percentages of Ly6+ cells, CD11b+ cells, and CD4+ cells, and reduced levels of IL-6, TLR4, INF-γ, iNOS, IL1-β, CCL-20, CXCL-1-2, CXCL-11 12; in CNS: reduced percentages of Ly6+ cells, CD11b+ cells, and CD4+ cells, and IL-1ra, IL-1α, IL-5, IL-7, IL-12, IL-15, IL-27, IL-28, TNF-α, G-CSF, osteopontin, VCAM-1, ICAM-1, pentraxin 3, CCL-5, CXCL-5, CXCL-10, CXCL-11, CXCL-13; inhibited oxidative stress and suppressed mitochondria damages in SC	EAE in C57BL/6N mice	[232]

Abbreviations: AD—atopic dermatitis; BALF—bronchoalveolar lavage fluid; CLP—cecal ligation and puncture; DNBS—2,4 dinitrobenzene sulfonic acid; DNCB—2,4-dinitrochlorobenzene; DAI—disease activity score; DSS—dextran sodium sulfate; EAE—experimental autoimmune encephalomyelitis; GSH—glutathione; HMBG1—High Mobility Group Box 1; HO-1—heme oxygenase; ICAM-1—intercellular adhesion molecule 1; JAK—Janus kinase; LNs—lymph nodes; LPS—lipopolysaccharide; MDA—malondialdehyde; MMP—matrix metalloproteinase; MPO—myeloperoxidase; NF-κB—nuclear factor kappa-light-chain-enhancer of activated B cell; Nrf2—nuclear factor erythroid 2-related factor 2; OA—osteoarthritis; OVA—ovalbumin; SC—spinal cord; SD—Sprague—Dawley; SOD—superoxide dismutase; STAT—signal transducer and activator of transcription; Treg—T regulatory cell; VCAM-1—vascular cell adhesion protein 1.

## Data Availability

As this article is a review, it does not present any new data that requires repository submission. All data analyzed and discussed in this review are derived from previously published studies, which are cited in the manuscript. Therefore, no new data were generated or analyzed in this study.

## Supplementary Materials

The following supporting information can be downloaded at https://www.mdpi.com/article/10.3390/antibiotics14070697/s1, Table S1: Plant species from which verbascoside has been isolated., Table S2: Antimicrobial activity of verbascoside.
