# Supplementary material for: Multifaceted Biological Properties of Verbascoside/Acteoside: Antimicrobial, Cytotoxic, Anti-Inflammatory, and Immunomodulatory Effects
Source: Antibiotics (Basel). 2025 Jul 11;14(7):697. doi: 10.3390/antibiotics14070697 (PMC12291726; doi:10.3390/antibiotics14070697)
Supplement: Supplementary file 1 [file antibiotics-14-00697-s001.zip › antibiotics-3707667-supplementary/Supplementary Table S1.pdf]

Supplementary files

Table S1. Plant species from which verbascoside has been isolated

Family	Plant species *	Plant part	Reference
Verbenaceae	*Aloysia polystachya* (Griseb.) Moldenke	Leaves	[12]
	Duranta repens L. (syn. *D. erecta* L.)	Aerial parts	[13]
	Lantana camara L.	Leaves	[14]
	Lantana lilacina Desf.	Leaves	[15]
	Lantana trifolia L.	Leaves	[16]
	Lippia alba f. *intermedia* Moldenke	Aerial parts	[17]
	Aloysia citriodora Palau *(syn. Lippia citriodora* Kunth)	Leaves	[18]
	Lippia dulcis Trevir	Flowers	[19]
	Lippia multiflora Moldenke	Leaves	[20]
	Lippia nodiflora	Whole plant	[21]
	Lippia triphylla (L. Hér) Kuntze	Leaves and stems	[22]
	Tectona grandis L.f.	Leaves	[23]
Lamiaceae	*Ajuga chamaepitys* (L.) Schreb.	Aerial parts	[24]
	Ajuga incisa	/	[25]
	Ajuga japonica	/	[25]
	Ajuga nipponensis	/	[25]
	Callicarpa macrophylla Vahl	Aerial parts	[26]
	Callicarpa peii Chang	Stems	[27]
	Clerodendron trichotomum Thunb.	Leaves	[11]
	Clerodendrum cyrtophyllum Turcz	Leaves	[28]
	Clerodendrum infortunatum L. (syn.: *C. viscosum* Vent.)	Leaves	[29]
	Clerodendrum inerme L. Gaertn (syn. *Volkameria inermis* L.)	Roots	[30]
	Colebrookea oppositifolia Smith	Roots	[31]
	Dracocephalum heterophyllum	Whole plant	[32]
	Lagopsis supina (Steph. ex Willd.) lk. -Gal. ex Knorr	Whole plant	[33]
	Leonurus glaucescens Bunge	Aerial parts	[34]
	Lepechinia speciosa (St. Hill) Epling	Aerial parts	[35]
	Leucoseptrum japonicum (Miq.) Kitamura et Murata	Roots	[36]
	Marrubium alysson L.	Aerial parts	[37]
	Marrubium deserti de Noé	Aerial parts	[38]
	Marrubium thessalum Boiss. & Heldr.	Aerial parts	[39]
	Marrubium vulgare L.	Aerial parts	[40]
	Nepeta ucrainica L.	Aerial parts	[41]
	Phlomis grandiflora H. S. Thompson var. *mbrilligera* (Hub.-Mor.) Hub.-Mor.	Aerial parts	[42]
	Phlomis amanica Vierch.	Aerial parts	[43]
	Phlomis armeniaca Willd.	Aerial parts	[44]
	Phlomis fruticosa L.	Aerial parts	[45]
	Phlomis integrifolia Hub.-Mor.	Aerial parts	[46]
	Phlomis lunariifolia Sm.	Aerial parts	[47]
	Phlomis longifolia Boiss. & El. var. l*ongifolia*	Aerial parts	[48]
	Phlomis monocephala P.H. Davis	Aerial parts	[49]

	Phlomis physocalyx Hub. Mor	Aerial parts	[50]
	Phlomis tuberosa L.	Aerial parts	[48]
	Phlomis viscosa Poiret	Aerial parts	[51]
	Salvia viridis L. cvar. Blue Jeans	Aerial parts	[52]
	Scutellaria indica	Whole plant	[53]
	Scutellaria lateriflora L.	Aerial parts	[54]
	Scutellaria prostrata Jacq. ex Benth	Aerial parts	[55]
	Scutellaria salviifolia Bentham	Aerial parts	[44]
	Sideritis cypria Post.	Flowers	[56]
	Stachys byzantina C. Koch.	Aerial parts	[57]
	Stachys germanica L. subsp. *salviifolia* (Ten.) Gams	Leaves and inflorescence	[58]
	Stachys iva Griseb.	Aerial parts	[59]
	Stachys lanata Crantz.	Aerial parts and roots	[60]
	Stachys lavandulifolia Vahl	Aerial parts	[61]
	Stachys macrantha (C. Koch) Stearn (syn. *Betonica grandijflora* Willd.)	Aerial parts	[62]
	Stachys officinalis Trevisan	Aerial parts	[63]
	Stachys sieboldii	Leaves	[64]
	Stachys schtschegleevii Sosn.	Stems	[65]
	Stachys tetragona Boiss. & Heldr. ex Boiss.	Aerial parts	[66]
	Teucrium hyrcanicum L.	Aerial parts	[67]
Oleaceae	*Abeliophyllum distichum* Nakai	Leaves and stems	[68]
	Fraxinus angustifolia Vahl	Galls	[69]
	Fraxinus excelsior L.	Galls; leaves	[69, 70]
	Forsythia viridissima Lindley	Leaves and fruits	[71]
	Forsythia koreana Nakai	Leaves	[71]
	Ligustrum lucidum W.T. Aiton	Fruit	[72]
	Ligustrum vulgare L.	Leaves	[73]
Orobanchaceae	*Castilleja linariaefolia* Benth.	Leaves, stems, roots	[74]
	Castilleja tenuiflora Benth. (synonyms: *C angustifolia* M. Martens & Galeotti, *C. canescens* Benth.)	Aerial parts	[75]
	Cistanche deserticola Y.C. Ma	/	[76]
	Cistanche tubulosa (Schenk) Wight	Aerial parts	[77]
	Cistanches salsa (C.A. Mey) G. Beck	Stems	[78]
	Lathraea squamaria L.	Aerial parts	[79]
	Orbanche rapum-genistae Thuill.	/	[8]
	Orobanche cernua Loefling	Whole plant	[80]
	Pedicularis chamissonis Steven	Leaves	[81]
	Pedicularis condensata Bieb.	Aerial parts	[82]
	Rehmannia glutinosa Libosch.	Leaves	[83]
Acanthaceae	*Acanthus hirsutus* Boiss	Aerial parts	[84]
	Acanthus ilicifolius var. *xiamenensis*	Whole plant	[85]
	Acanthus mollis L.	Leaves	[86]
	Blepharis ciliaris (L.) B.L. Burtt.	Aerial parts	[87]
	Blepharis edulis (Forssk.) Pers.	Aerial parts	[88]
	Odontonema cuspidatum (Nees) Kuntze	Aerial parts	[89]
	Strobilanthes cusia (Nees) Kuntze	Aerial parts	[90]

Plantaginaceae	*Linaria corifolia* Desf.	Aerial parts	[91]
	Penstemon barbatus (Cav.) Roth	Leaves	[92]
	Penstemon hirsutus L. (Willd.)	Leaves	[93]
	Plantago asiatica L.	Aerial parts	[94]
	Plantago depressa	Leaves	[95]
	Plantago holosteum Scop.	Aerial parts	[96]
	Plantago lanceolata L.	Inflorescence	[97]
	Plantago major L.	Aerial parts	[98]
	Plantago media	Leaves	[95]
	Plantago psyllium	Seeds	[99]
Scrophulariaceae	*Brandisia hancei* Hook. f.	Stems and leaves	[100]
	Buddleja brasiliensis Jacq. ex Spreng	Dried roots and aerial parts	[101]
	Buddleja davidii Franch	Leaves	[102]
	Buddleja macrostachya Benth.	Aerial parts	[103]
	Buddleja nitida Benth.	Leaves	[102]
	Buddleja officinalis Maxim.	Flower buds, inflorescences	[104]
	Buddleja salviifolia (L.) Lam.	Leaf	[105]
	Euphrasia rostkoviana Hayne	Aerial parts	[106]
	Leucophyllum frutescens	Aerial parts	[107]
	Odontites serotina (Lam.) Dum.	Whole plant	[108]
	Paulownia tomentosa (Thunb.) Steud. var. *tomentosa*	Bark	[109]
	Scrophularia ningpoenis Hemsl	Root	[110]
	Scrophularia striata Boiss.	Aerial parts	[111]
	Verbascum mucronatum Lam.	Flowery parts	[112]
	Verbascum ovalifolium Donn ex Sims	Aerial parts	[113]
	Verbascum speciosum	Aerial parts	[114]
	Verbascum xanthophoeniceum Griseb.	Aerial	[115]
Gesneriaceae	*Conandron ramnoidioides*	Whole plant	[116]
	Lysionotus pauciforus Maxim.	Aerial parts	[117]
	Moussonia deppeana (Schldl. & Cham) Hanst	Aerial parts	[118]
	Oreocharis auricula (S. Moore) C. B. Clarke	Aerial parts	[119]
Bignoniaceae	*Adenocalymma marginatum* DC.	Stems and roots	[120]
	Amphilophium paniculatum (L.) Kunth	Leaves	[121]
	Arrabidaea harleyi A.H. Gentry	Bark	[122]
	Arrabidaea pulchra (Cham.) Sandwith	Leaves	[123]
	Bignonia binata Thunb. (syn. Clytostoma binatum Thunb.)	Leaves	[124]
	Campsis grandiflora (Thunb.) K. Schum (syn. *C. chinensis* (Lam.) Voss)	Flowers, leaves	[125]
	Incarvillea compacta Maxim.	Whole plant	[126]
	Incarvillea younghusbandii Sprague	Roots	[127]
	Jacaranda caucana Pittier subsp. *sandwithiana* A.H.Gentr	Stems	[128]
	Jacaranda cuspidifolia Mart.	Bark	[129]
	Jacaranda mimosifolia D. Don	Leaves	[130]
	Macfadyena unguis-cati (L.) A.H. Gentry	Aerial parts	[131]
	Markhamia tomentosa (Benth.) K. Schum. Ex Engl.	Leaves - ident	[132]
	Markhamia lutea Seemann ex Baillor	Roots	[133]
	Markhamia platycalyx (Baker) Sprague	Leaf	[134]

	Oroxylum indicum (L.) Kurz	Stem bark	[135]
	Pithecoctenium crucigerum (L.) A.H. Gentry	Stems	[136]
	Tecoma stans (L.) Juss. ex Kunth	Leaves	[137]
Pedaliaceae	*Harpagophytum procumbens* DC.	Secondary roots	[138]
	Sesamum indicum L.	Leaf, stem, root, flower	[139]

* The names of plant species are provided as reported in the literature
/—Part of the plant not specified
