# Supplementary material for: Multifaceted Biological Properties of Verbascoside/Acteoside: Antimicrobial, Cytotoxic, Anti-Inflammatory, and Immunomodulatory Effects
Source: Antibiotics (Basel). 2025 Jul 11;14(7):697. doi: 10.3390/antibiotics14070697 (PMC12291726; doi:10.3390/antibiotics14070697)
Supplement: Supplementary file 1 [file antibiotics-14-00697-s001.zip › antibiotics-3707667-supplementary/Supplementary Table S2.pdf]

**Supplementary files**

**Table S2.** Antimicrobial activity of verbascoside.

| Antimicrobial activity | Microorganisms / mechanisms of action / type of antimicrobial assay | Verbascoside (V) / isoverbascoside (iV) concentration | Ref. |
|---|---|---|---|
| Antibacterial activity | *Staphylococcus aureus* | MIC 512 μg/mL (V) | [112] |
| | | MIC 63 μg/mL (V) | [164] |
| | | MIC 62.5 μg/mL (V) | [105] |
| | | MIC 400 μg/mL (V) | [166] |
| | | MIC >200 μg/mL (V) | [168] |
| | | MIC 600 μg/mL (iV+V) | [122] |
| | *S. aureus* (MRSA) | MIC 128 μg/mL (V) | [167] |
| | *S. aureus* (MRSA) | MIC >200 μg/mL (V) | [168] |
| | *S. aureus* (MDR) | MIC 625-2500 μg/mL (V) | [155] |
| | *Staphylococcus epidermidis* | MIC 32 μg/mL (V) | [164] |
| | *Streptococcus pyogenes* | MIC 62 μg/mL (V) | [164] |
| | *Enterococcus faecalis* | MIC 256 μg/mL (V)data | [112] |
| | | MIC 600 μg/mL (iV+V) | [122] |
| | *Micrococcus luteus* | MIC 300 μg/mL (iV+V) | [122] |
| | *Bacillus subtilis* | MIC 125 μg/mL (V) | [105] |
| | | MIC 600 μg/mL (iV+V) | [122] |
| | *Bacillus mycoides* | MIC 600 μg/mL (iV+V) | [122] |
| | *Mycobacterium smegmatis* | MIC >600 μg/mL (iV+V) | [122] |
| | *Escherichia coli* | MIC 512 μg/mL (V) | [112] |
| | | MIC 125 μg/mL (V) | [105] |
| | | MIC 600 μg/mL (iV+V) | [122] |
| | | MIC >200 μg/mL (V) | [168] |
| | *Escherichia coli* O157:H7 and O111 | MIC ≤78.12 -10000 μg/mL (V) | [172] |
| | *Klebsiella pneumoniae* | MIC 62.5 μg/mL (V) | [105] |
| | *Serratia marcescens* | MIC 600 μg/mL (iV+V) | [122] |
| | *Proteus mirabilis* | MIC 128 μg/mL (V) | [167] |
| | *Pseudomonas aeruginosa* | MIC 256 μg/mL (V) | [112] |
| | | MIC >600 μg/mL (iV+V) | [122] |
| | *P. aeruginosa* (MDR) | MIC 625-2500 μg/mL (V) | [155] |
| Antifungal activity | *Candida albicans* | MIC 256 μg/mL (V) | [112] |
| | | MIC 125 μg/mL (V) | [177] |
| | | MIC 0.7-1.5 μg/mL (V) | [5] |
| | | MIC 0.7-3 μg/mL (iV) | [5] |
| | | MIC >12.5 μg/mL (V) | [178] |
| | *Candida kruzei* | MIC 256 μg/mL (V) | [112] |
| | | MIC 125 μg/mL (V) | [177] |
| | | MIC 1.5 μg/mL (V, iV) | [5] |
| | | MIC >12.5 μg/mL (V) | [178] |
| | *Candida parapsylosis* | MIC 256 μg/mL (V) | [112] |
| | | MIC 125 μg/mL (V) | [177] |
| | | MIC 1.5 μg/mL (V, iV) | [5] |
| | | MIC >12.5 μg/mL (V) | [178] |
| | *Candida tropicalis* | MIC 1.5 μg/mL (V) | [5] |
| | | MIC 6 μg/mL (iV) | [5] |

| | | | |
|---|---|---|---|
| | | MIC >12.5 μg/mL (V) | [178] |
| | *Candida guilliermondii* | MIC 0.7 μg/mL (V) | [5] |
| | | MIC 1.5 μg/mL (iV) | [5] |
| | *Candida glabrata* | MIC >12.5 μg/mL (V) | [178] |
| | *Cryptococcus neoformans* | MIC 15.6 μg/mL (V) | [177] |
| | | MIC >12.5 μg/mL (V) | [178] |
| | *Aspergillus fumigatus, A. flavus, A. niger, A. parasiticus* | MIC >12.5 μg/mL (V) | [178] |
| Antprotozoal activity | *Leishmania amazonensis* (promastigote) / arginase inhibition | $EC_{50}$ 19 μM (V) | [6] |
| | *Leishmania amazonensis* (amastigote) / arginase inhibition | $EC_{50}$ 32 μM (V) | [184] |
| | *Leishmania donovani* (amastigote) | $IC_{50}$ 8.7 μg/mL (V)<br>$IC_{50}$ 9.2 μg/mL (iV) | [186] |
| | *Leishmania infantum* | $IC_{50}$ > 64 μM (V) | [189] |
| | *Trypanosoma brucei rhodesiense* (trypomastigote) | $IC_{50}$ 14.2 μg/mL (V)<br>$IC_{50}$ 6.2 μg/mL (iV) | [186,187] |
| | *Trypanosoma brucei* | $IC_{50}$ > 64 μM (V) | [189] |
| | *Trypanosoma cruzy* (trypomastigote) | $IC_{50}$ > 90 μg/mL (V)<br>$IC_{50}$ > 90 μg/mL (iV) | [186,187] |
| | | $IC_{50}$ 32.81 μM (V) | [189] |
| | *Plasmodium falciparum* | $IC_{50}$ > 50 μg/mL (V)<br>$IC_{50}$ 37.5 μg/mL (iV) | [186,187] |
| | | $IC_{50}$ > 64 μM (V) | [189] |
| Antiviral activity | SARS-CoV-2 / in silico study of enzyme inhibition (methyltransferase, helicase, $Pl^{pro}$, $M^{pro}$, RdRp) | n.a. | [194,197,198] |
| | SARS-CoV-2 / in vitro antiviral assay on Vero-E6 cells | $IC_{50}$ 118.3 *μg* /mL | [194] |
| | HSV-1 / in vitro antiviral assay on Vero cells | $EC_{50}$ 58 μg/mL | [35] |
| | HSV-2 / in vitro antiviral assay on Vero cells | $EC_{50}$ 8.9 μg/mL | [35] |
| | RSV / in vitro antiviral assay on HEp-2 and A549 cells | $EC_{50}$ 15.64 ± 1.07 ng/mL<br>$CC_{50}$ 740.34 ± 8.23 ng/mL | [201] |
| | Influenza virus (A/FM/1/47 H1N1, FM1) / *in vivo* antiviral assay in C57BL/6 and Balb/c mice | 80 mg/kg | [157] |
| | VSV NJ strain / in vitro antiviral assay on WISH cells | n.a. | [157] |
| | Dengue virus / in vitro antiviral assay on Vero and LLCMK2 cells | $EC_{50}$ 3.4 ± 0.4 μg/mL | [202] |

Abbreviations: MIC- minimum inhibitory concentration; $EC_{50}$-ffective concentration value; $IC_{50}$-inhibitory concentration; $CC_{50}$-cytotoxic concentration, MDR-multidrug-resistant; MRSA-methicillin-resistant *S. aureus*
